# Mobile Apps to Improve Brace-Wearing Compliance in Patients with Idiopathic Scoliosis: A Quality Analysis, Functionality Review and Future Directions

**DOI:** 10.3390/jcm12051972

**Published:** 2023-03-02

**Authors:** Han Eol Cho, Chan Woong Jang, Sung Rae Cho, Won Ah Choi, Jung Hyun Park

**Affiliations:** 1 Department of Rehabilitation Medicine, Rehabilitation Institute of Neuromuscular Disease, Gangnam Severance Hospital, Yonsei University College of Medicine, Seoul 06229, Republic of Korea; 2 Department and Research Institute of Rehabilitation Medicine, Yonsei University College of Medicine, Seoul 03722, Republic of Korea; 3 Brain Korea 21 PLUS Project for Medical Science, Yonsei University College of Medicine, Seoul 03722, Republic of Korea; 4 Graduate Program of Biomedical Engineering, Yonsei University College of Medicine, Seoul 03722, Republic of Korea; 5 Department of Integrative Medicine, Yonsei University College of Medicine, Seoul 06229, Republic of Korea; 6 Department of Medical Device Engineering and Management, Yonsei University College of Medicine, Seoul 06229, Republic of Korea

**Keywords:** braces, mobile applications, patient compliance, scoliosis, smartphone

## Abstract

This study was performed to review which mHealth apps that improve brace-wearing compliance are currently available, and to carry out their quality assessments by listing their functionalities. We found ten mHealth apps in the literature review and commercial mHealth apps market (Google Play and App store). Then, the quality of these apps was evaluated by their transparency, health content, excellent technical content, security/privacy, issues of usability, and subjective ratings (THESIS) scale, and the functionalities of the included apps were reviewed. Regarding these functionalities, four categories (data acquisition, compliance enhancement, educational components, and additional functionalities) and twelve subcategories were identified. The mean overall quality of the apps was 3.00 out of 5. Although four of the apps achieved a score of 3.0 or more for their overall quality, indicating an acceptable quality, none of the apps scored higher than 4.0, which indicated a high or excellent quality. According to the sections, the transparency section had the highest rating (3.92) and the security/privacy section received the lowest rating (2.02). Given that the overall quality of current mHealth apps was not high, and their potential to motivate patients with idiopathic scoliosis to adhere to their bracing treatment, it is necessary to develop high-quality apps with appropriate functionalities for supporting brace treatment.

## 1. Introduction

Scoliosis is defined as a torsional deviation from the normal growth of the spine and trunk, and is generally diagnosed with a Cobb angle >10° [1]. When there is no other underlying disease that can be identified, scoliosis is denoted as idiopathic. Idiopathic scoliosis (IS) is one of the most common musculoskeletal disorders related to the spine, with a prevalence of 2–4% in the general population, and progresses the most at the beginning of puberty [1,2]. The standard management for IS comprises three categories: observation, bracing, and surgical treatment [3]. Observation is indicated for a mild curvature of less than 20°, and bracing is especially recommended for curves between 20° and 40° [4,5]. Exercise is also recommended as a standalone treatment or as an addition to bracing for preventing the progression of scoliosis in mild to moderate IS patients [6]. If the curve of the spine exceeds 40°, surgical treatment is considered [7].

Non-surgical treatments, including bracing, have been widely used and have emerged as proven methods to treat IS [2,5]. Despite previous studies that have demonstrated the effectiveness of brace treatment with a high level of evidence [6,8,9], there is a poor compliance in patients who wear a brace, which is found to range from 33–75% over the prescribed time, and is a problem to be solved [10,11,12]. To overcome this, different efforts have been conducted to help patients adhere better to bracing treatments, and the improved monitoring of brace wearing has been proposed to enhance brace compliance [10,13].

Mobile health (mHealth) can be helpful in this area [14]. Due to its widespread use, practicality, and portability, the mobile phone is one of the tools that is mostly utilized to deliver mHealth. In this context, mHealth applications (apps) or health-related smartphone apps have been used to assist with the healthcare of patients with chronic diseases [15,16,17]. mHealth apps have functionalities to monitor symptoms, collect real-time data, and provide feedback through graphical materials to users, so they have the potential to be helpful for the self-management of diseases [18,19].

Despite the rapid progression of IS in adolescence and its obvious need for self-management, including bracing, and the increase in the smartphone usage of this age group, no studies have assessed mHealth apps to support brace wearing compliance in patients with IS. Thus, the main aim of this study was to conduct a systematic review and evaluation of the mHealth apps that were focused specifically on IS patients’ compliance to bracing. Thus, we identified which mHealth apps are currently developed and available, and carried out a quality assessment of them with validated rating scales, providing the functionalities of these apps.

## 2. Materials and Methods

### 2.1. Search and Selection Strategy

To find mHealth apps that can improve the brace adherence of IS patients, based on the PRISMA-ScR guidelines, we conducted comprehensive searches of literature databases and commercial mobile app markets. First, we used five electronic databases, including PubMed, Embase, the Cochrane Library, Scopus, and the Institute of Electrical and Electronics Engineering (IEEE) Xplore. After trying several combinations of words and expressions, on 20 June 2022, the databases were searched with the following search terms: “scoliosis” AND “brace”* AND “mobile” AND (“application”* OR “app”*); “scoliosis” AND “brace”* AND “mhealth” OR “m-health”; “scoliosis” AND “brace”* AND “smartphone”; and “scoliosis” AND “brace”* AND “mobile phone”. A total of two reviewers independently reviewed the titles and abstracts of the articles to find studies to be excluded (Table 1). Then, the full text of the remaining articles was reviewed. The references of the retrieved articles were also hand-searched, and any additional eligible studies were included. After a full text review, all the mHealth apps associated with improving the compliance of IS bracing covered in these articles were included for analysis.

Then, the Google Play store and the App Store, the most popular smartphone app stores, were searched with the keyword “scoliosis” on 20 June 2022, to carry out the mobile app markets review. The resulting apps were collected. An initial screening based on the name and description of the apps in the app market, after removing any duplicates, was performed by two independent reviewers. The eligible apps were downloaded and installed onto each appropriate device for a full review. The inclusion and exclusion criteria are also shown in Table 1. If cross-platform duplicates were found, only the most recently updated version of the app was included for analysis.

### 2.2. In-Depth Analysis and Procedure

We extracted the basic characteristics of the apps (Supplementary Table S1). The medical professionals’ involvement in their developing processes and contents was determined through examining their descriptions on the app store and associated websites. Then, for quality assessment, the included mHealth apps were scored using the transparency, health content, excellent technical content, security/privacy, issues of usability, and subjective ratings (THESIS) scale, which was developed by Levine et al. for evaluating the mHealth apps within a marketplace, by the two independent authors [20]. The detailed structure of the scale can be seen in Supplementary Table S2.

Prior to rating the apps, the two reviewers were trained in the use of the THESIS scale and used the included apps for a minimum of 10 min. The information available through an internet search for the app and its associated websites was additionally referenced. Each item was scored using a five-point scale, and we calculated the mean for each item to combine the two reviewers’ individual ratings. Then, a mean score was given for each section. Finally, the mean values of the 6 sections were computed to give an overall rating of the app quality. We set 3.0 as the cut-off level for the minimum acceptability score. The app is classified as high quality if its rating is 4.0 or more, and low quality if it is less than 2.0.

In addition, the functionalities of the apps were summarized by the two authors. Based on the types of supports to improve brace adherence provided by these apps, each author assigned names to the functionalities. They then got together to confirm the extracted functionalities for each app. The retrieved functionalities were sorted into different groups based on their similarities. Finally, four themes of the functionalities were grouped. Any discrepancies between the two reviewers in the categorization of the functionalities were resolved through continued discussion between them.

## 3. Results

### 3.1. Search Results

**Figure 1:**
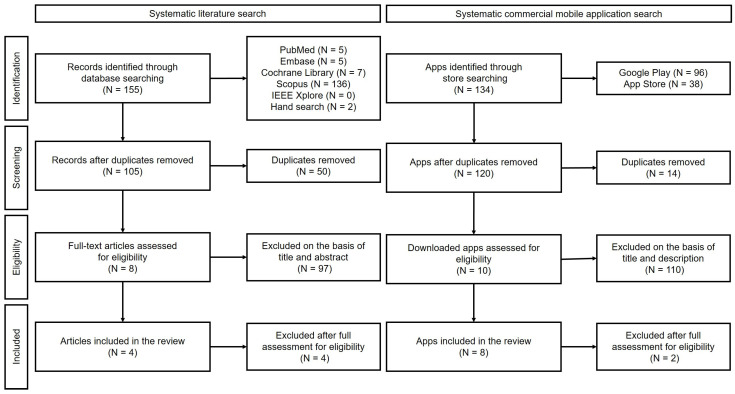
Flowchart of included apps selection processes.

A total of 105 non-duplicated articles were collected from the initial search, of which eight articles were eligible for full-text screening based on the title and abstract. After full-text screening, three apps from three articles remained: the BeMobil Orthotimer [21,22] and the Pain Monitor and WeChat Mini Program [23,24] apps (Figure 1).

The results of the commercial mobile app markets search yielded a total of 120 apps, after excluding any duplicates. After screening, eight eligible apps remained, and each was fully analyzed (Figure 1). BeMobil Orthotimer was included in both the literature and the app store screening. Ultimately, ten apps were included in the final analysis.

### 3.2. Quality by the THESIS

**Figure 2:**
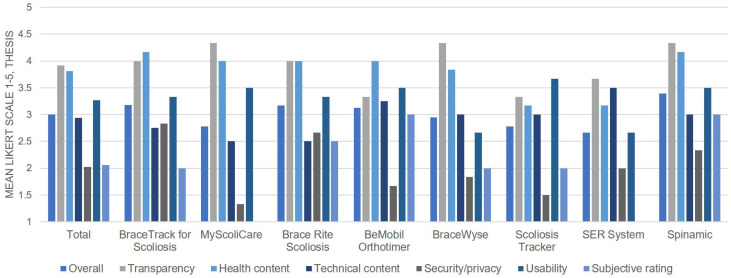
Quality ratings of commercial mHealth apps by the THESIS.

Figure 2 shows the quality ratings of the commercial mHealth apps via the THESIS scale. Two of the apps found in the literature search, the Pain Monitor and WeChat Mini Program, were not rated, because these were not exclusively developed for patients with scoliosis [23,24]. The Cohen weighted kappa coefficient indicated a good agreement between the two reviewers, showing that the interrater reliability was 0.90.

The mean overall app rating of all the apps was 3.00 out of 5 (95% confidence interval [CI], 2.83–3.18). The most highly rated app was the Spinamic, and its mean overall score was 3.38. The worst rated app was the SER system, and its mean overall score was 2.67. None of the apps scored higher than 4.0, which indicated a high or excellent quality. According to the sections, the transparency section had the highest rating (3.92; 95% CI, 3.62–4.21). The security/privacy section received the lowest rating: 2.02 out of 5 (95% CI, 1.64–2.40).

### 3.3. Functionality

The functionalities of the included apps are summarized in Table 2.

#### 3.3.1. Data Acquisition

Data acquisition refers to the function of collecting information related to scoliosis severity and brace wearing. Our review identified two (20%) apps, Scoliosis Tracker and Spinamic, that had the function of checking scoliosis severity. Scoliosis Tracker provides a digital scoliometer for patients to track their degree of exacerbation every day. This app visualizes the changes according to growth in a graph so that it can be recognized in a simple way. Spinamic has the function to directly enter the start, angle, and end of the curvature, but these data cannot be tracked.

Depending on whether the brace wearing time was entered into the app manually or automatically, the function could be divided. There were five (50%) apps, BeMobil Orthotimer, BraceWyse, Brace Rite Scoliosis, and SER system in which data were automatically collected. Meanwhile, WeChat Mini Program could collect data automatically, but the patient should upload the brace wearing result manually through a button click. These apps were remotely linked to sensors through Bluetooth or Near-Field Communication (NFC). All the sensors used, except for WeChat Mini Program, were temperature-sensitive sensors, and the sensor connected to the BeMobil Orthotimer app had the additional functions of detecting body humidity, mechanical pressure, and acceleration. The sensor connected to the WeChat Mini Program was a mechanical pressure-sensitive one. The remaining four apps required the user to enter the wearing time themselves, or press the start and end buttons to record their wearing time.

#### 3.3.2. Compliance Enhancement

The compliance-enhancing function was subdivided into four subcategories: goal setting, data visualization, motivation, and testimonial and practical tips. Features that set a goal for the wearing time were included in two (20%) apps, BraceTrack for Scoliosis and Brace Rite Scoliosis, which allow users to enter their target wearing time on an hourly basis. In addition, these apps can inform the patient of the remaining time the brace needs to be worn that day by subtracting the wearing time from the set target time.

Except for two apps, Scoliosis Tracker and Pain Monitor, the remaining eight apps (80%) showed the wearing time to the user in the form of a graph or text. A total of four apps offered motivation to wear the brace with text messages; “Good Start. Keep Bracing!” for BraceTrack for Scoliosis users every time they used the app, “More hours to reach today’s goal!” for Brace Rite Scoliosis, and “Important! Make sure to use this checklist every day.” for Scoliosis Tracker. Patients used Pain-Monitor-received notifications to daily answer questions on the app and were judged on their compliance to brace wearing based on the answers to these questions (e.g., “When have you been able to wear the brace since you went to bed yesterday?”). In total, two apps, MyScoliCare and Spinamic, motivated users by giving them points according to their time spent brace wearing. In addition, in Spinamic, users could receive an alarm at four set times a day as a reminder to wear the brace.

Some apps provided sharing information capabilities by providing patient testimonials or practical tips for managing scoliosis; the “Pinboard” section for BeMobil Orthotimer and “Patient Stories” for Scoliosis Tracker.

#### 3.3.3. Educational Components

Overall, five of the ten (50%) apps provided educational information. In more detail, three of the ten (30%) apps provided general information on scoliosis, such as conservative and surgical treatment types, and diagnostic criteria. BeMobil Orthotimer had a quiz function that patients could use to improve their adherence in long-term treatment. Brace Rite Scoliosis had links to educational resources which are hosted on websites operated by the Texas Scottish Rite Hospital for Children. These contained information such as definitions, causes, diagnostic criteria, and treatment options for scoliosis. Scoliosis Tracker featured an “Education” section in the app that contained 16 subsections, covering definitions, diagnostic criteria, treatment options, and accounts of the daily lives of patients.

To provide physiotherapeutic scoliosis-specific exercises (PSSE), MyScoliCare had three different sections including “Record Exercise Video”, “Setup Exercise Plan”, and “Start Exercise Session” that could be used by patients using the Schroth method for their treatment. The SER Systems app gave audio and visual feedback of the PSSEs conducted while wearing the brace.

#### 3.3.4. Additional Functionalities

There were three apps (30%) that provided exports of brace wearing times; a comma-separated value format in BeMobil Orthotimer and BraceWyse, and a portable document format in BraceTrack for Scoliosis. Direct communication functions allow users to send patient- or brace-related information to the developers of the apps or the patient’s physicians. Through this function, the user may be able to solve the inconveniences of using the app or of wearing the brace as soon as possible. In addition, these three apps had the ability to remind users of their next appointment with medical staff.

## 4. Discussion

This is the first study to comprehensively review the mHealth apps for improving the brace compliance of IS patients that are available to use, and to thoroughly assess their quality using a validated rating scale, or the THESIS scale. We included ten mHealth apps from the literature and commercial app markets, and found that these apps have diverse functionalities (4 categories and 12 subcategories) and a minimum acceptable quality, but that they are not high-quality apps.

The brace wearing time is a known important factor that determines the effect of brace treatment. The longer the wearing time of the brace, the more the progression of scoliosis is prevented [8,9,25]. Additionally, the higher the brace compliance, the significantly lower the amelioration of the Cobb angle and Perdriolle degree [26]. As if to reflect this, most apps had the functionality of data acquisition of the brace wearing time (80%) and four of the (40%) apps could automatically check the wearing time and import data. Contrarily, conventional brace treatment for IS, including compliance monitoring, has been quite patient-dependent. Even if the patient reports that he wore a brace well, there is no way to check whether the patient is really adhering or not [27]. Indeed, the majority of patients are known to wear their brace for less than half of the prescribed amount of time [25]. With the method of informing patients that their compliance was being electronically monitored, brace adherence could be increased [13]. Therefore, mHealth apps that check the brace wearing time and induce compliance in the brace wearing, such as through functional feedback, including giving rewards to patients, could be the solution.

In the method of automatically checking the brace wearing time, some apps used pressure or temperature sensors. Sensing technology is advancing quickly today [28,29]. It means that the use of sensor-based real-time monitoring of bracing is expected to expand further. For the pressure sensor especially, the development of various telecommunication technologies, including Bluetooth or NFC, would be another possibility to accelerate the development of monitoring technologies. Although the principle of action of the scoliosis brace has been based on the action of three-point pressure, the actual corrective effects in relationship to the applied pressures and brace wearing time have not yet been thoroughly investigated. Thus, the development of mHealth apps with the ability to monitor real-time applied pressure and wearing time is needed to improve the precision of brace treatment for IS patients, and to evaluate its long-term clinical effects.

In addition to monitoring the brace wearing time, frequent communication between physicians and patients with actual compliance data has been proven to increase brace adherence [30]. From this point of view, to improve compliance, it may be helpful to use the functionalities offered by the existing apps, such as visualizing the changes in the wearing time, providing messages encouraging brace use, and awarding scores based on the wearing time. Another useful alternative is to provide patients with daily direct feedback by measuring the progression of the spinal curvature change. Thus, the feedback that enhances patients’ insight has shown some feasibility and effectiveness for the management of brace usage in IS patients [31]. Some apps had tried to check changes of spinal curvature by using a digital scoliometer. However, the accuracy of the digital scoliometer is still controversial. There is no evaluation method yet that can replace radiologic evaluations [32]. We believe that these issues might be solved by linking the radiologic data taken at hospitals directly with the apps, or by developing new diagnostic tests using an mHealth app, which can be implemented by patients themselves in future.

mHealth apps can be useful tools that can provide appropriate information about PSSE, which has been proven to be effective for treating adolescent IS patients with mild and moderate curves [33,34,35,36]. PSSE slows the progression of scoliosis and/or reduces the curve severity measured by the Cobb angle. However, there were only a few apps that contained actual educational components that included PSSE. A total of two of the eight apps that were analyzed provided information on PSSE by providing simple exercise videos. Accordingly, when creating mHealth apps for scoliosis, it is essential to include comprehensive educational information on precise exercise techniques tailored to patients’ severity. Furthermore, incorporating motion detection and real-time coaching functions into the apps is expected to improve the effectiveness of exercise education [37].

Our data showed that the quality of the existing commercial mHealth apps was not good enough, and varied across the evaluation domains. Our data showed that the health content sections including items “Appropriate measurement” and “Appropriate interpretation of data”, which are key functions related to improving the brace wearing compliance, were rated as a high quality, while “Quality of information” and “Presentation of information” were scored relatively low. In other words, these findings suggest that the mHealth app has the potential to develop further by enhancing the functions in low-quality domains. In addition, mHealth apps should be more considerate of non-English speakers and those with a lower health literacy. It is particularly important for developers to consider that the mHealth apps that dealt with personal health information scored low in the section of security/privacy.

This study has some limitations. First, we did not purchase or use the associated braces and sensors in order to find out how effectively they were able to measure the wearing time; therefore, we could not draw any conclusions concerning the efficacy of these apps. Second, unfortunately, as we did not gather any user opinions or experiences regarding the included apps, patient compliance (both objective and subjective) and clinical availability could not be accurately determined. This information could help with the overall evaluation of these apps and the production of future apps. Third, the search and inclusion strategy may have missed potentially relevant apps and articles. For example, there may be apps in non-English languages or in the non-English literature, or listed in apps stores outside those included in this study.

## 5. Conclusions

In conclusion, each mHealth app employed several strategies to improve brace wearing, including a sensor-based automatic brace wear checking system, approaches for assessing the degree of scoliosis, and methods for offering suitable exercise suggestions. These functionalities have a large amount of room for improvement, and we believe that mHealth apps will be a great tool in increasing IS patients’ compliance with brace treatment. Thus, it is necessary to develop appropriate and diverse functionalities through apps of a high quality, and improve them using continued research. This development will establish a new paradigm for brace treatment for IS.

## Figures and Tables

**Table 1:** Inclusion and exclusion criteria for commercial mHealth apps and articles in the final review.

**Mobile Application Market Review**	
**Inclusion Criteria**	**Exclusion Criteria**
Available to the public (with or without payment) Self-contained product Centered on supporting brace treatments for patients with scoliosis	Irrelevant to scoliosis Only provided general information about scoliosis Focused only on the diagnosis or surgical treatment of scoliosis Developed for healthcare professionals
**Literature Review**	
**Inclusion Criteria**	**Exclusion Criteria**
Available in full text Address patients with scoliosis Refer to the development and use of mobile apps as tools specific to monitoring brace wearing compliance	Not available in full text Not centered on patients with scoliosis Did not involve any types of digital tools Refer to digital tools in different contexts, such as developing sensors, using as digital scoliometers, and designing braces, which were not directly related to the monitoring of brace compliance Ongoing research

**Table 2:** Summary of functionalities provided by mHealth apps.

App Name	BeMobil Orthotimer	BraceTrack for Scoliosis	BraceWyse	Brace Rite Scoliosis	MyScoliCare	Scoliosis Tracker	SER System	Spinamic	Pain Monitor	WeChat Mini Program
Data acquisition										
Scoliosis severity check	.	.	.	.	.	V	.	V	.	.
Brace wearing time import	Automatic	Manual	Automatic	Automatic	Manual	Manual	Automatic	Manual	.	Manual
Brace connection with sensors	NFC ^1^	.	NFC ^2^	Bluetooth ^2^	.	.	Bluetooth ^2^	.	.	Bluetooth ^3^
Compliance enhancement										
Goal setting	.	V	.	V	.	.	.	.	.	.
Data visualization	V(graph)	V(graph + calender)	V(graphs)	V(graphs)	V(text)	.	V(graph)	V(graph)	.	V
Motivation	.	V(text)	.	V(text)	V(point)	V(text)	.	V(point,notify)	V(notify)	.
Testimonial and practical tips	V	.	.	.	.	V	.	.	.	.
Educational components										
General information of scoliosis	V(quiz)	.	.	V	.	V	.	.	.	.
PSSE	.	.	.	.	V(Schroth)	.	V	.	.	.
Additional functionalities										
Brace wearing time export	V(CSV)	V(PDF)	V(CSV)	.	.	.	.	.	.	.
Direct communication	.	V	V	.	.	V	.	Unknown	.	V
Appointment reminder	V	.	.	.	V	V	.	.	.	.

App, application; CSV, comma separated value; PDF, portable document format; PSSE, physiotherapeutic scoliosis specific exercises; and NFC, Near-Field Communication. ^1^ Connected with a sensor sensing temperature, body humidity, mechanical pressure, and acceleration. ^2^ Connected with a sensor sensing temperature. ^3^ Connected with a sensor sensing mechanical pressure.

## Data Availability

Data are available on request due to restrictions. The data presented in this study are available on request from the corresponding author.

## Supplementary Materials

The following supporting information can be downloaded at: https://www.mdpi.com/article/10.3390/jcm12051972/s1, Table S1: Summary of basic information of included mHealth apps; Table S2: Detailed app rating items of the THESIS.
