# Supplementary material for: Mobile Apps to Improve Brace-Wearing Compliance in Patients with Idiopathic Scoliosis: A Quality Analysis, Functionality Review and Future Directions
Source: J Clin Med. 2023 Mar 2;12(5):1972. doi: 10.3390/jcm12051972 (PMC10003789; doi:10.3390/jcm12051972)
Supplement: Supplementary file 1 [file jcm-12-01972-s001.zip › jcm-2212612-supplementary.pdf]

Supplementary Table S1. Summary of basic information of included mHealth apps.

App name	BeMobil Orthotimer	BraceTrack for Scoliosis	BraceWyse	Brace Rite Scoliosis	MyScoliCare	Scoliosis Tracker	SER system	Spinamic	Pain Monitor	WeChat (Mini Program)
Developer	nova motum Services & Consulting Gmb1ya	BraceTrack, LLC	BraceWyse (Yuchen Sun)	Texas Scottish Rite Hospital for Children	BA Winston	BLUE LINER MARKETING, LLC	Scoliosistoday, LLC	Value and Trust Co., Ltd.	Azucena Garcia-Palacios	WeChat International Pte. Ltd.
-Provider involvement	Yes	No	No	Yes	No	Yes	Unknown	Yes	Yes	Yes
Operating system	Android, iOS	Android, iOS	Android, iOS	Android, iOS	Android, iOS	iOS	iOS	iOS	Android, iOS	Android, iOS
Version	1.0.8	1.4.6	32	0.5.6	5	1.1	0.4.1	1.03	1.8	8.0.18
-Upload, yr	2019	2019	2021	2021	2019	2019	2021	2018	2017	2011
-Last update, yr	2020	2021	2021	2021	2019	2019	2022	2019	2020	2022
Cost	Free	Free	Free	Free	Free	Free	Free	Free	Free	Free
-In app purchase	Yes ($36.99)	No	No	No	No	No	No	No	No	No
-Sensors purchase	Needed*	No	Needed†	Needed‡	No	No	Needed[b]	No	No	Needed[c]
Target users	Patients	Patients	Patients +Caregivers	Patients +Caregivers	Patients +Caregivers	Patients +Caregivers	Patients	Patients	Patients	Patients

* Sensor sensing temperature, body humidity, mechanical pressure, acceleration.

† Sensor sensing temperature.

‡ Sensor sensing mechanical pressure.

Supplementary Table S2. Detailed app rating items of the THESIS.

Section	Item	Detail
**Transparency**		
	Cost of app	Are the prices, subscriptions, and in-app purchases accurately conveyed?
	Consent	What is the quality of the consent process, if any?
	Accuracy of app store description	How accurate is the app store description of the app's purpose?
**Health content**		
	Appropriate measurement	Does the app appropriately measure what it claims to measure?
	Appropriate interpretation of data	Does the app appropriately interpret what it claims to interpret?
	Quality of information	How optimal is the quality of information?
	Potential for harm	Is the potential for harm minimized?
	Literacy level	How appropriate is the literacy level for the app's intended audience?
	Presentation of information	Is information presented in an optimal manner? For example, is scaffolding used?
**Technical content**		
	Software performance/stability	Does the app run well with zero interface crashes or bugs?
	Interoperability	Is the app able to exchange information with EHRs and other apps?
	Bandwidth	Does the app require significant bandwidth to run?
	Application size	Does the app require significant storage capacity?
**Security/Privacy**		
	Protection against theft and viruses	Does the app follow best practices in security with optimal anti-virus and safeguards against breaches?
	Authentication	Is the authentication procedure optimal?
	Data sharing	When sharing information, does the app use best practices?
	Maintenance	Does the app have regular cycles to update and patch its security?
	Signaling of breaches	If a breach occurs, does the app have a method to notify its users?
	Anonymization	Does the app appropriately anonymize individuals?
**Usability**		
	Installation and setup	How would you rate are installation and setup?
	Functionality	Quality of ease of use, navigation, gestural design, help/instructions?
	Aesthetics	Quality of layout, graphics, visual appeal, and image readability?
	Customization/tailoring	Ability to customize and tailor to the specific user's needs?

	Ease of use for users with low literacy and numeracy	Is the app usable by users with low literacy and numeracy?
	Availability in multiple languages	Is the app available in multiple languages?
**Subjective**		
	Recommend app	Would you recommend this app
	Overall star rating	What is your overall rating of this app?
